# Virginia Society of Surgeon Dentists

**Published:** 1842-12

**Authors:** 


### Miscellaneous Notices.

*Virginia Society of Surgeon Dentists.*—In a letter just received from our friend, Dr. J. D McCabe, of Richmond, Va., we are informed that a convention of surgeon dentists had recently been held in that city, which resulted in the organization of the above named society. We had before been apprised of the intention of some of the dentists of Virginia to hold a convention for this purpose, and could we have spared the time, we should, in compliance with an invitation which we received, have been present on the occasion. We are pleased with the move that has been made to elevate the character of the profession in Virginia, and to protect its citizens against the impositions that are daily being practised upon them by ignorant and unprincipled charlatans. We hope the example will be followed by dentists in other states, and that the work of reform may not cease until the profession is thoroughly purged from the quackery with which it now abounds.

With most of the gentlemen composing the Virginia Society of Surgeon Dentists, we are personally acquainted, and believe them to be well read and skilful practitioners. Many of them are members of the American Society of Dental Surgeons.

It will be perceived from the following proceedings of the Society, which Dr. McCabe had the kindness to enclose to us, that it is their intention to apply to the Legislature of the State for an act of incorporation. We wish them success in their noble undertaking, and should the Legislature of Virginia act in this matter, and that they will, we cannot doubt, we hope that every dentist who shall be permitted to practise in the state, will hereafter be required to undergo a rigid examination before a board of examiners appointed by the Society, and obtain from them a license. Let none but such as are thoroughly qualified be permitted to practice.

We hope the dentists in Western Virginia will co-operate with those already belonging to the Society, and should a law be passed, requiring of all who shall hereafter practise in the state, a license from the Society, we would suggest the propriety of having two boards of examiners, one in the eastern and one in the western part of the State.—Eds.

